# Babchi Oil-Based Nanoemulsion Hydrogel for the Management of Psoriasis: A Novel Energy Economic Approach Employing Biosurfactants

**DOI:** 10.3390/gels8120761

**Published:** 2022-11-23

**Authors:** Aftab Alam, Mohammed H. Alqarni, Ahmed I. Foudah, Mohammad Raish, Mohamad Ayman Salkini

**Affiliations:** 1 Department of Pharmacognosy, College of Pharmacy, Prince Sattam Bin Abdulaziz University, Al Kharj 11942, Saudi Arabia; 2 Department of Pharmaceutics, College of Pharmacy, King Saud University, Riyadh 11451, Saudi Arabia

**Keywords:** Babchi oil, nanoemulsion-based hydrogel, nanoemulgel, low energy emulsification, psoriasis

## Abstract

The current research aimed to assess the Babchi oil nanoemulsion-based hydrogel prepared using biosurfactants through a low-energy emulsification process for the topical management of psoriasis. The emulsification capacity and solubilities of many nanoemulsion constituents such as surfactants, co-surfactants, and oil were considered to determine the range of concentration of the constituents. Pseudoternary phase diagrams were created using the method of titration. Nanoemulgel structure, morphology, micromeritics, conductivity, and viscosity were all optimized. The assessment of the Babchi oil nanoemulgel included particle size, polydispersity index (PDI), drug content, pH, spreadability, rheological management, ex vivo drug study, 2,2-diphenyl-1-picrylhydrazyl (DPPH) scavenging ability, in vitro drug release, release kinetics, and dermatokinetics. The selected ratios of the surfactant mixture (Smix) taken were 3:1. The entrapment efficiency estimated was 91.298%. The zeta potential of Babchi oil was observed to be −24.93 mV at 25 °C with water as a dispersant, viscosity as 0.887 cP, and material absorption as 0.01 nm. The size distribution of the particle was 108 nm by the intensity and the conductivity observed was 0.03359 mS/cm. The cumulative amount of Babchi oil penetrated and fluxed by nanoemulgel was considered larger (*p* ≤ 0.05) than the conventional formulations. Skin retention was observed to be good with decreased lag time. The formulation followed the Higuchi Korsmeyer for Fickian Peppas model for in vitro drug release studies. The oil was most effective on the epidermal layer of the skin for treatment. It was established that the Babchi oil nanoemulgel formulation had superior permeability capabilities for topical and transdermal administration and is a viable alternative to traditional formulations.

## 1. Introduction

Psoriasis is an inherited skin condition that presents with persistent non-contagious intense itching. Symptoms of this condition include deformation, inflammation, and scaly and thickened skin [1]. The global statistics of recorded cases of psoriasis are about two to five percent [2,3,4]. The condition progresses in many ways, and its differentiation is noted by the undergoing inflammation, rash localization, irritation of the local area, intensity of other traits, and its occurrence. This condition is classified into four types: erythroderma, pustular, and guttate, along with a persistent plaque. It manifests during the early forties and is observed equally in both genders [5,6]. Longer periods of therapy are required to treat psoriasis as it is a chronic disorder. Cyclosporines, methotrexate, and retinoids are the commonly used anti-psoriatic medications. Still, several adverse effects occur due to these drugs that include renal and liver dysfunction, loss of hair, stomach discomfort, and inflammation of lips [1,7]. 

Approved topical therapies for the treatment of psoriasis, such as dithranol, emollients, and coal tar, are usually safe; however, they are minimally effective. Medical advancements are exploring several methods for the treatment of this condition, such as steroidal lotions, oral and injectable medications, etc. Although these medications provide momentary relief, they do not treat the symptoms as a whole because they lack a secure and optimum vehicle that can properly transport the anti-psoriatic properties of the drug for maximum treatment benefit [4,8]. To overcome the limits of traditional therapies, researchers have turned to nanoscience and nanomedicines to improve the effectiveness and lessen the undesirable adverse effects of anti-psoriatic medications. These medicines have become valuable because of their increased bioavailability, low prescription doses, and nanosized delivery of drugs. Similar to these methods, nanoemulsion is also the technique that employs a colloidal approach to administering nanosized particles of active drug moieties to the afflicted skin areas as the drugs have large surface areas.

A nanoemulsion is a transparent, stable kinetic dispersion of two insoluble phases of oil and water in the presence of surfactant particles ranging in size from 5 to 200 nm [9,10]. The usage of nanoemulsion as a vehicle for anti-psoriatic medications is favourable since it does not exhibit flocculation, internal creaming, deposition, or coarsening, which are prevalent in macroemulsions [11]. The ability to permeate is strong in nanoemulsions, along with increased drug-loading potential when used topically [4,12]. The choice of the right surfactants and oils is critical for an optimum nanoemulsion. The potential usage of biosurfactants in substitution of synthesized equivalents is considered better in regards to expense and price, durability, and compatibility with the environment. Biosurfactants are agents that are active on the surface and provide a natural alternative to their chemical equivalents. These are less harmful and also perform well during high temperature and pH conditions. Biosurfactants are significantly durable as bacterial growth is quick, and the source is numerous and producible [13]. Hydrogels are a potential type of material made from natural or artificial polymer that has three-dimensional network structures with large water content [14]. It is the most ideal biomaterial for the creation of surface coatings in the prevention and treatment of multi-drug resistant infections due to their high hydrophilicity, complex three-dimensional network, cell adhesion, and distinctive biocompatibility [15,16,17,18,19,20]. 

Plant-derived essential oils are fragrant liquids that have high volatility. Their abundant chemicals, such as hydrocarbons, terpenoids, coumarins, and phenols, are observed and used for their range of therapeutic properties [21]. Babchi oil produced from *P. coryfolia* (*Psoralea coryfolia* L.) is widely recognized among essential oils for its antioxidant, antibacterial, anticancer, immunomodulatory, anti-inflammatory, and antifungal effects [22]. The herb is found to grow annually in warm climates in countries such as China, India, South Africa, and Pakistan. It has become a standard treatment in the Chinese and Ayurveda fields of therapy [23].

Babchi has been used to treat a range of skin ailments, including psoriasis, leukoderma, and leprosy [24]. Isopsoralen, psoralen, and bakuchiol are the main components obtained from Babchi oil and are recognized for their various properties. Although the benefits of Babchi oil are abundant, it has poor physical qualities such as degradation susceptibility, sensitivity to light, hydrophobicity, and extreme viscosity, which has reduced the practical usage of the oil and has been confined to medicines. Recent trends in using Babchi oil include formulations such as emulgel [25] and other forms; however, formulation of the oil into a nanoemulsion-based hydrogel has not been reported. This study aims to investigate Babchi oil nanoemulsion-based hydrogel or nanoemulgel with low energy emulsification method for the management of psoriasis.

## 2. Results and Discussion

### 2.1. Component Screening

For development of the system for nanoemulsion for delivering the drug transdermally, certain factors, such as the solubility of the drug and its components, are required for the drug to reach and permeate within the skin. The solubility of Babchi oil was investigated.

### 2.2. The Behavior of Phase and Nanoemulsion Optimization

**Figure 1:**
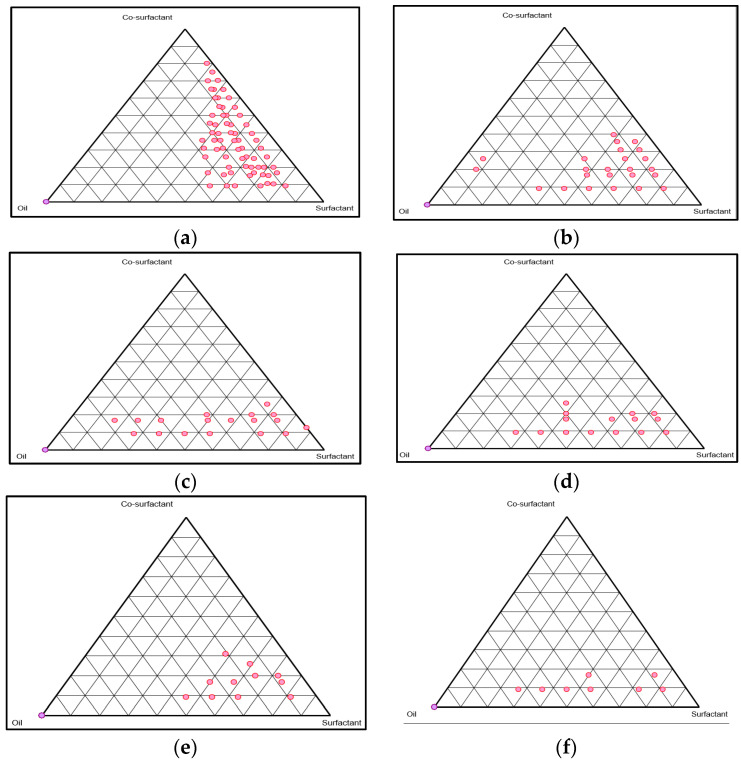
Pseudoternary phase diagrams displaying the area of nanoemulsion of Smix. (**a**) 3:1 (Selected ratio for Smix). (**b**) 4:1. (**c**) 5:1. (**d**) 2:1. (**e**) 1:0. (**f**) 1:1.

Constructing diagrams for pseudoternary phases is required because they help in finding the existing nanoemulsion ranges. Diagrams of the phase represent the translucent regions of nanoemulsion. Visual observation of the other regions on the diagram of the phases is of traditional and turbid type [26]. Separate ratio diagrams for the surfactant to co-surfactant of the pseudoternary phase were constructed to determine the regions of O/W of nanoemulsion and its formulations for optimization as shown in Figure 1.

### 2.3. Characterization: Structure and Morphology

**Figure 2:**
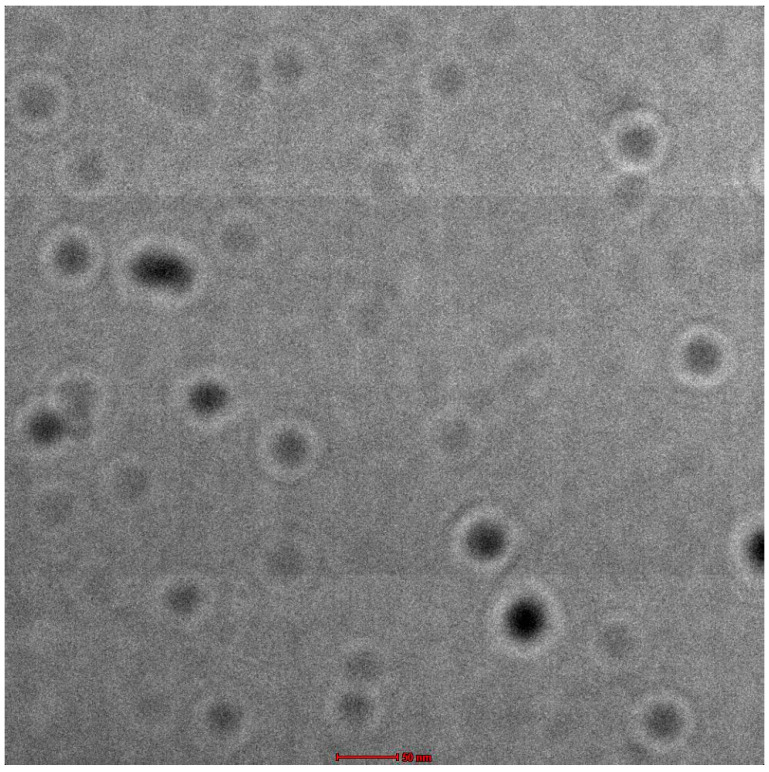
TEM measurement (scale set at 50 nm).

Nanoemulsion appeared to be dark around the other light surroundings when observed positively under a TEM (transmission electron microscope). The average size of the droplet was smaller than that of 100 nm from the sample. The droplets were observed to be within the range of ≤200 nm of nanosize, and therefore the emulsion that was prepared was considered to be nanoemulsion. Figure 2 shows the TEM of the spreadability of Babchi oil nanoemulgel.

### 2.4. Micromeritics

To determine the effective and safer dose, it is crucial to characterize the nanoemulsion sizes [27]. It was found that the current formulations fell into the nanosize range. This was also observed through the decreased values of the index of polydispersity. The index for polydispersity is defined as the SD (standard deviation) ratio to the mean size of droplets and denotes the homogeneity of the size inside the formulation. 

### 2.5. Determination of Conductance and Viscosity

The nanoemulsion viscosities were determined to be minimal, with a range of 9.8 ± 0.42 to 36.1 ± 0.63 mPaS. This behaviour of nanoemulsions demonstrated that they were unsuitable for topical applications. Therefore, it justified the integration of nanoemulsion within the matrix of gel, resulting in a high viscosity value in the formulated nanoemulgel. The nanoemulsion conductance was observed to be high with a value of 48.2 ± 0.03 to 161.1 ± 0.13 μs/cm which resulted in the formulation being of the nanoemulsion O/W category.

### 2.6. Nanoemulgel Formulation

Even though the formulations were discovered to be in the range suitable for nanosize, their poor viscosities hampered their application and were thus deemed unsuitable for cutaneous usage. To improve the nanoemulsion application, respective viscosities were increased by inserting nanoemulsions within the gel matrix of 940 carbomers, leading to formations of nanoemulgel that were appropriate, homogenous, yet extremely viscous enough to be dermally administered.

#### 2.6.1. Nanoemulgel pH Determination and Concentration of Drug

Values of pH for all nanoemulgels separately were observed at 6.5 ± 0.15 to 6.9 ± 0.47 in the neutral range. This allowed the formulated mixture to be used on the skin easily.

#### 2.6.2. Ability to Spread

Due to the utilization of the formulated mixture, the spreading ability of nanoemulgel preparation was assessed. Its texture is pleasant for skin inflammation as it distributes quickly, with maximal drag and slip. The spreading ability of the formulations was determined to be from the 5.6 ± 0.21 to 6.2 ± 0.12 gcm/S ranges. The increased radius indicates a greater ability to spread.

#### 2.6.3. Measurement of Rheological Behaviour

The ability to spread and flow along with the release of oil is all governed by rheological properties. The release of oil and its substances through the formulated mixture is primarily regulated by the components present in it. The index used for measuring the consistency was 1 s^−1^ at the shear rate and it equaled the apparent viscosity. The index of consistency of the formulated mixture was observed to be 0.33. The index of flow was defined as the measurement of the system’s divergence from Newtonian behavior (n = 1). The value of “n” lesser than 1 implies pseudoplastic flow or narrowing of shear, whereas a value greater than 1 suggests a narrowing of shear or dilatants. The index of flow indicated the ability to flow the formulated mixture from the container.

In general, a lesser index of flow results in a thick base. The index of flow for nanoemulgel was 0.33, presenting characteristics of pseudoplastic flow. Pseudoplasticity is usually caused by a network of colloidal structures which align themselves in the shear direction. This lowers the viscosity and increases the rate of shear. The flow characteristic of pseudoplastic nature validates the designed system’s need to exert some force of expulsion.

### 2.7. Applying Central Composite Rotatable Design for Formulation

#### Effect of Independent Variables on Particle Size (nm), Zeta Potential, and Time Phases

**Figure 3:**
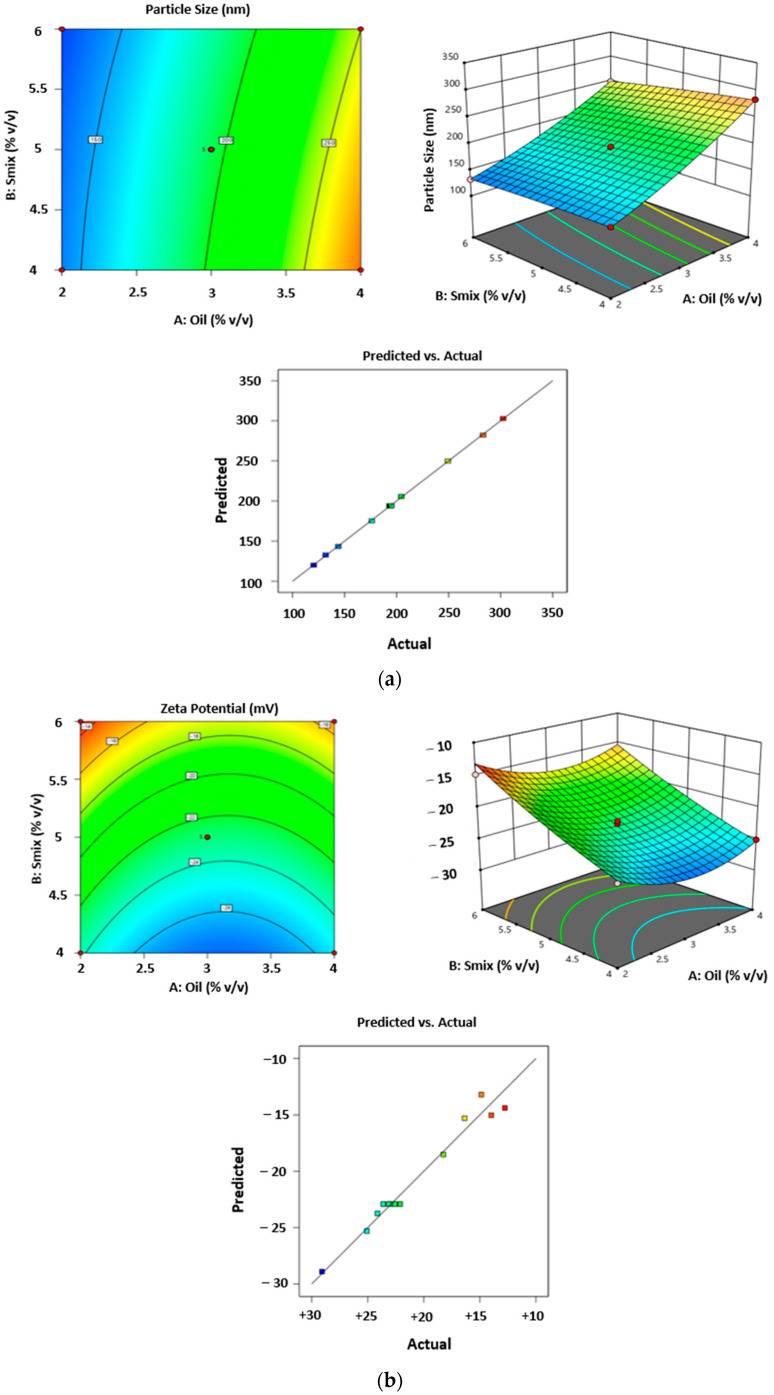

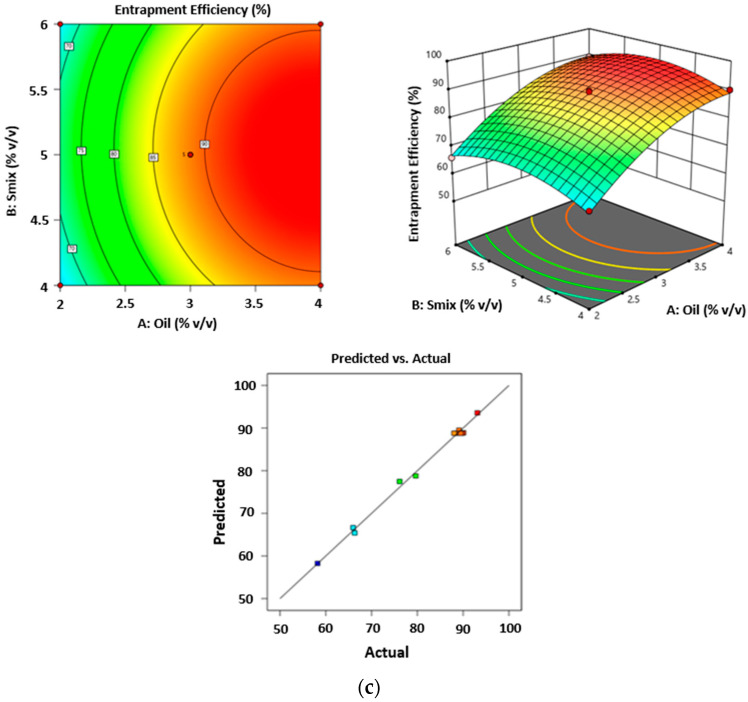
3D Response surface graphs showing the effect of oil and Smix concentration on (**a**) Particle Size (nm), (**b**) Zeta Potential (mV) and (**c**) Entrapment Efficiency (%EE).

**Figure 4:**
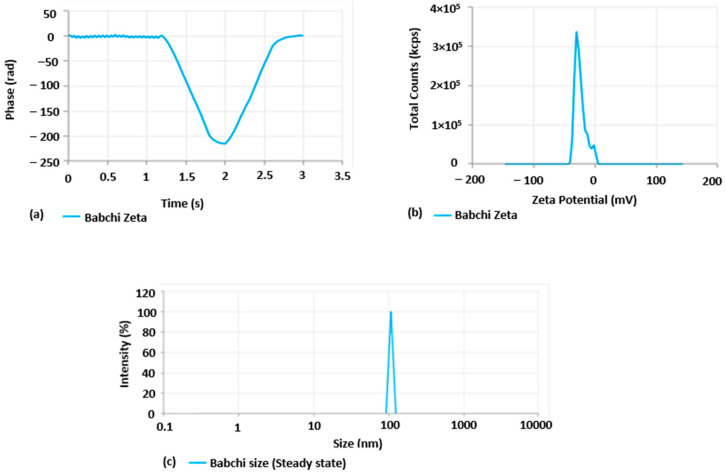
Zeta potential data analysis, (**a**) phase–time graph of Babchi oil zeta potential, (**b**) total count of Babchi oil zeta potential distribution graph, (**c**) Babchi size distribution and intensity graph.

Three-dementional response surface graphs present the effect of Babchi oil and Smix concentration on particle size (nm), zeta potential (mV), and entrapment efficiency of 91.298% EE and are presented in Figure 3 and Table 1. The zeta potential of Babchi oil was observed to be −24.93 mV at 25 °C with water as a dispersant, viscosity as 0.887 cP, and material absorption as 0.01 nm as observed in Figure 3a,b. The zeta potential distribution conductivity observed was 0.03359 mS/cm. Figure 3c shows the size distribution of 108 nm by intensity. Figure 4a depicts the phase time graph of zeta potential and Figure 4b,c depict the total counts of zeta potential distribution graph and Babchi size distribution and intensity graph, respectively.

Particle size (nm) (Y1) = 194.03 + 63.96A − 10.75B − 5.40AB + 9.80A^2^ − 1.80B^2^;

Zeta Potential (mV) (Y2) = −22.91 − 0.91A + 5.13B − 0.13AB + 2.89A^2^ + 0.63B^2^;

Entrapment Efficiency (%) (Y3) = 88.78 + 11.59A + 0.46B − 0.15AB − 5.84A^2^ − 5.33B^2^.

### 2.8. Studies for Permeability of Skin

Babchi oil nanoemulgel formulation had higher drug profile transfer throughout the skin of rats with *p* < 0.0001 and flux of 4.21 ± 2.25 μg/cm^2^/h compared to conventional formulation with the flux of 3.06 ± 3.12 μg/cm^2^/h. The presence of Babchi oil in the nanoemulgel system might contribute to the significant disparity in the percentage of drug distribution. The Babchi oil permeability was increased by enhancing the solubility of the surfactant during the formulation. Due to the compact design of the developed systems, they were capable of deeper penetrating the intrinsic layers of skin and resulting in greater absorption of the drug. In comparison to standard formulations, nanoemulgel was proven to improve penetration rates within deep layers of skin and also reduce lag time. The total amount of oil penetration, skin retention, flux, lag time, LAE, and enhancement ratio was determined. 

### 2.9. In Vitro Release Studies

**Figure 5:**
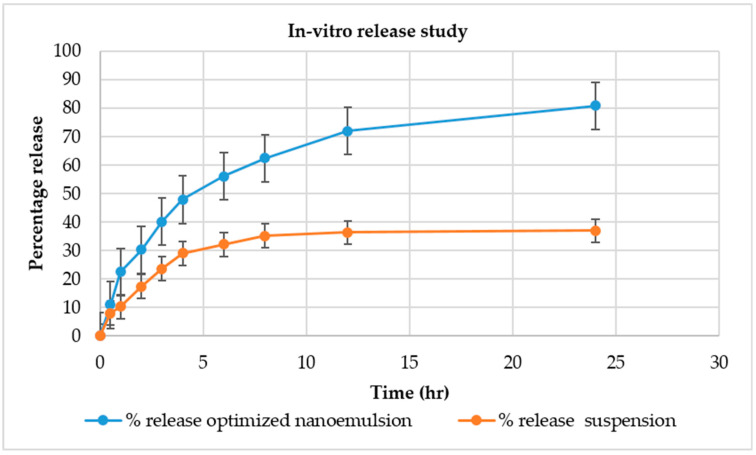
In vitro release study of optimized nanoemulsion and release suspension.

**Figure 6:**
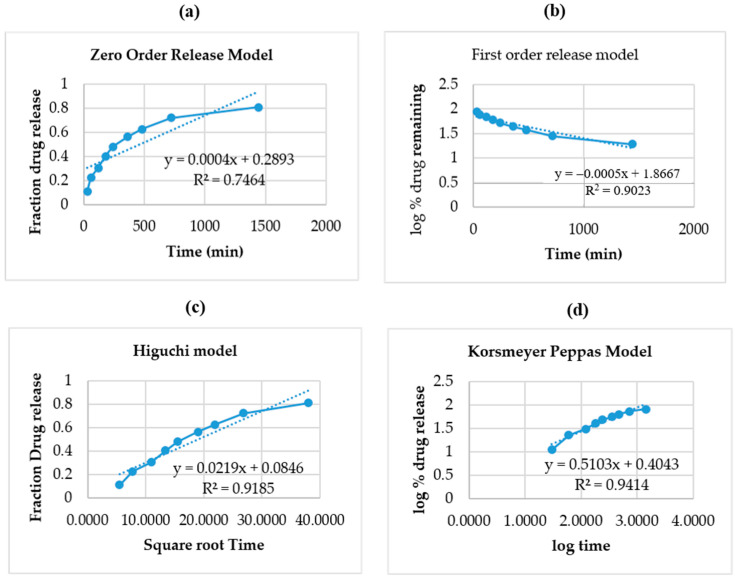
Different models used for in vitro release studies: (**a**) Zero order release model (**b**) First order release model (**c**) Higuchi model (**d**) Korsmeyer Peppas model.

**Figure 7:**
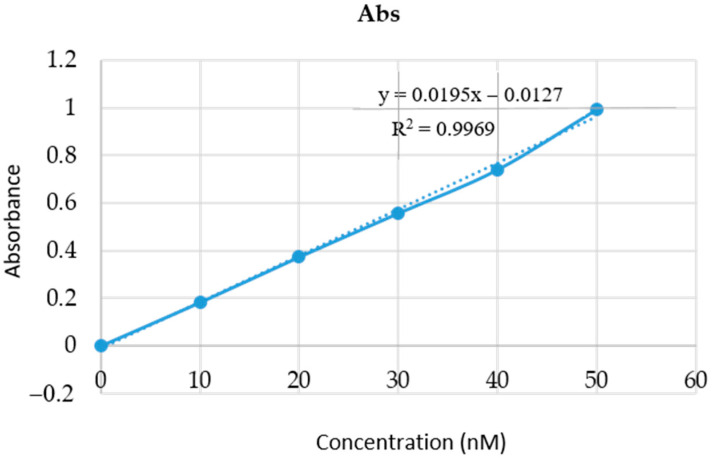
Absorption observed at different concentrations of nanoemulgel.

**Figure 8:**
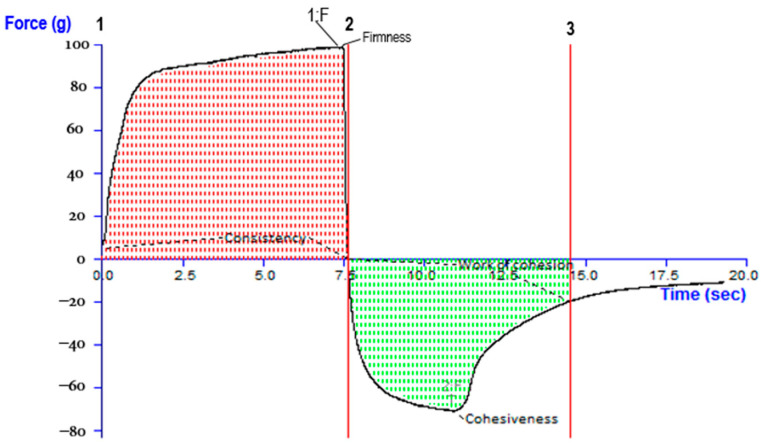
Release kinetics of nanoemulgel observed.

The nanoemulgel formulation was compared with the conventional formulation to observe the percentage of drug release in the epidermal layer. Figure 5 depicts the results obtained during the in vitro release study of the optimized nanoemulsion and its suspension. It was observed that the percentage of release from the optimized nanoemulsion was greater than 81.26 in comparison to the suspension of 38.19. It was observed that Babchi oil nanoemulgel produced a higher percentage of drug release in comparison to the conventional formulation and constantly increased with time. The different models used for the analysis of in vitro release such as the zero-order release model as depicted in Figure 6a produced higher effects and also increased with time. The y value taken was 0.0004x + 0.2893 and the R^2^ value taken was 0.7464 for plotting the graph. The first order release model was plotted with a y value of −0.0005x + 1.8667 and an R^2^ value of 0.9023 which is depicted in Figure 6b. It was observed that analysis with this model decreased the log percentage of the remaining drug with time. Figure 6c shows the Higuchi model shown with a y value of 0.0219x + 0.0846 and an R^2^ value of 0.9185. It was observed that this analysis model increased the fraction of drug release with time. Finally, the Korsmeyer Peppas model was implemented with a y value of 0.5103x + 0.4043 and an R^2^ value of 0.9414 and was plotted in Figure 6d. It was observed that Babchi oil nanoemulgel followed the Korsmeyer Peppas model and produced the most significant results of drug release with time. Higuchi model was considered the best with the Korsmeyer Peppas model producing the most potent diffusion. Figure 7 depicts the results obtained for studies of absorption (nM). The percentage of drug release by time graph of the nanoemulsion formulation was plotted and depicted in Figure 8.

### 2.10. DPPH Scavenging Activity of Babchi Oil Nanoemulgel

The lowering property of DPPH produced by the antioxidant was measured by observing a decrease at 517 nm of absorbance and the colorless occurrence of color violet was noted. Babchi oil nanoemulgel’s potential for the activity of antioxidants was reduced a little compared to the conventional formulation. The Babchi oil nanoemulgel produced an antioxidant activity of 69.1% while the conventional formulation produced 91.25%. Through these values, the potential for antioxidant activity of Babchi oil nanoemulgel was demonstrated as it did not change through entrapment within the formulation.

### 2.11. Dermatokinetic Study

**Figure 9:**
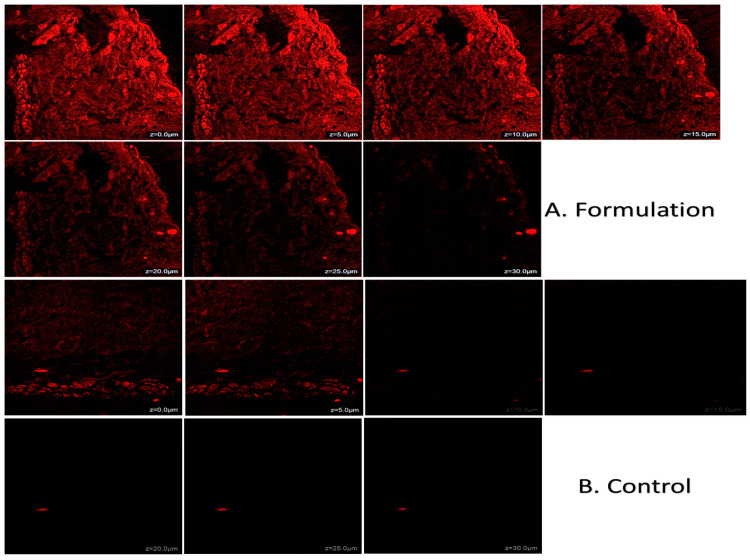
Images of CLSM within the perpendicular cross-section of optimal skin surface of rats.

**Figure 10:**
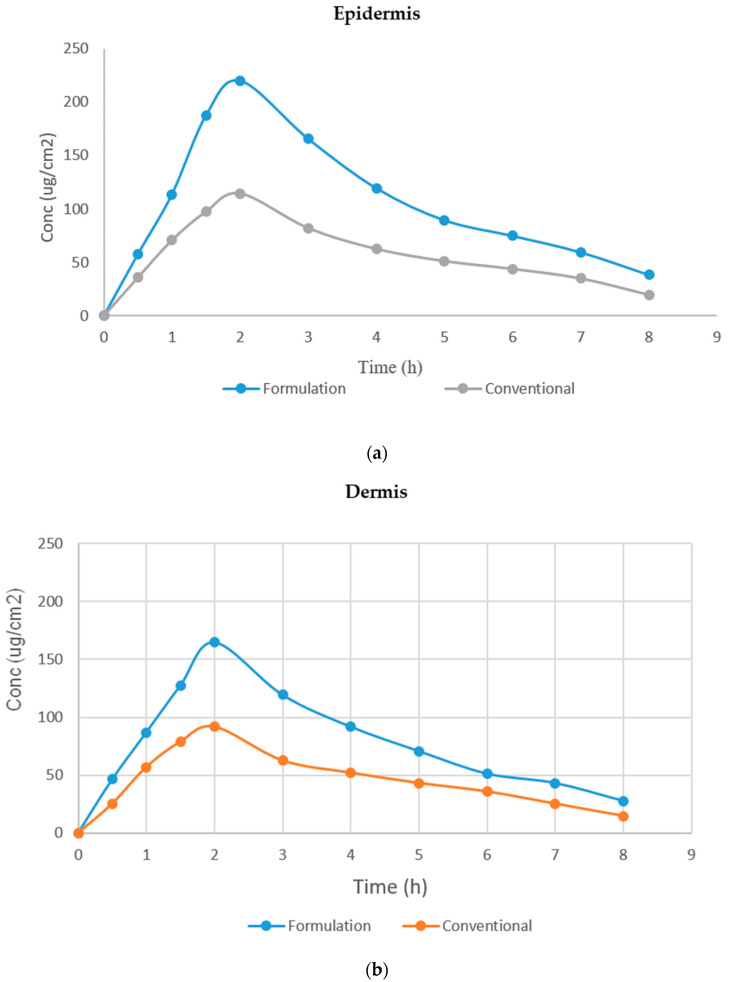
(**a**) Effect of Babchi oil nanoemulgel on epidermis concentration; (**b**) Effect of Babchi oil nanoemulgel on dermis concentration.

The quantity of Babchi oil present in the epidermis and dermis on rat skin following the application of the nanoemulgel formulation at different intervals of time was analyzed by ANOVA for statistical analysis to compare the epidermal and dermal layers of rat skin. Table 2 depicts the dermatokinetic study chart. It was revealed that the nanoemulgel formulation had a higher value of C skin max with 220.049 ± 5.00 μg/cm^2^ while the conventional medicine was lower on the epidermis. It was observed that the conventional formulation produced a higher value of C skin max with 375.918 ± 11.00 μg/cm^2^ in comparison to the nanoemulgel formulation on the dermis. The area under the curve (AUC) of the formulation was highest in the epidermal layer of the nanoemulgel formulation with a value of 872.4898 μg/cm^2^ while the conventional formulation presented with a value of 114.569 μg/cm^2^. The dermis layer presented a higher value of conventional formulation with the value of 375.918 μg/cm^2^ while the nanoemulgel produced only 164.875 μg/cm^2^. The Ke value nanoemulgel formulation on epidermal and dermal layers was 0.1329 μg/cm^2^ and 0.1393 μg/cm^2^, respectively, while the conventional formulation produced 0.1377 μg/cm^2^ and 0.1391 μg/cm^2^, respectively. This suggested better penetration of nanoemulgel formulation and its elasticity along with the activator when the nanoemulgel is applied topically. Figure 9 depicts the images obtained through CLSM within the perpendicular cross-section of optimal skin surface of rats while Figure 10a,b depicts the effects of Babchi oil nanoemulgel on epidermis and dermis concentration, respectively.

### 2.12. Studies for Stability

When the nanoemulgel formulations were centrifuged, no drug precipitation or phase separation was detected, indicating that the produced nanoemulgel was physically stable. Although these were exposed to thaw or freeze cycles, they did not exhibit symptoms of cracks or breaks. The findings of stability investigations demonstrated that nanoemulgels remain transparent, even 3 months later at different exposed temperatures such as 25 °C ± 2 °C, 37 °C ± 0.1 °C, along with 4 ± 0.2 °C. Even during stability investigation, each of the formulated mixtures was observed to be stable in terms of pH (6.8), transparency, the content of oil, and separation of phases. The increased viscosity of nanoemulgel may potentially limit Brownian mobility.

Nanoemulgels have great capacities for solubilization and for boosting permeability; due to this, nanoemulgel was used as a vehicle for distributing drug transdermally [28]. Aside from these facts, a topical approach for administration provides the opportunity to avoid the issues related to persistent oral dosing. The innovative techniques of nanoemulgel composed of combinations of aqueous and gel-based, surfactant and co-surfactants gels along with oil create multiple components for the drug loading system. Therefore, the nanoemulsions are created by determining the range of concentrations of each component. The size of the droplet, the conductance of the nanoemulsion, viscosity, and structural morphology were developed and optimized. Images observed through the TEM indicated a round shape. The formulations observed were in the range of 10–100 nm nano size. PDI value showed the droplets to be of consistent size throughout the formulations. O/W structure was observed which validated the nanoemulsion for its increased values of conductivity. Values obtained for viscosity were increased which made it suitable for skin application. The nanoemulsions that were induced within the base of the gel resulted in the formulation of the nanoemulgel. These nanoemulgel formulations had minimal loss of Babchi oil molecules. The pH of the formulation was similar to the skin pH, and therefore the formulation presented to be of minimal irritation in nature. The drag and slip phenomena with increased diameters were observed when formulations spreadability was checked. Higher viscosity content was observed along with a pseudoplastic manner when produced by the rheogram which ensured the flow would not occur within the system that was developed. In addition, collapsible tube and container filling would have required an ejection yield value [29]. 

The nanoemulgels formulated underwent studies for permeability in which Babchi oil nanoemulgel proved to be of higher significance as the permeation observed was higher and the components of the formulation correlated with each other. When the co-surfactant was reduced and the content of oil was increased, the permeability of the formulation was observed to be greater. Babchi oil and its permeability to penetrate the lipid bilayers of the skin occur due to the synergistic effect as it disrupts the order and improvises the thermodynamic effect of the formulation due to lesser concentration of surfactant in the skin [30]. Through this study, we also observed the co-surfactant effect when used as an enhancer for permeation when compared with the formulations that were optimized and those that were prepared without the usage of co-surfactants as the amount of drug permeation was low cumulatively during the flux. It was also observed that retention of formulation on the skin was improved along with the lag time. In comparison with the parameters for permeability, the optimized Babchi oil nanoemulgel had increased permeation of drug amount along with the flux and ratio for enhancement and high retention by the skin than the other plain and marketed formulations. The formulation’s AUC was higher in the epidermis, indicating improved formulation absorption and flow ability.

In this study, the nanoemulgel formulated presented with good potential for permeation without any induction of chemically synthesized enhancers. This reduced the cause of skin irritations and hence, the novelty of the study lies here because the constituents such as surfactant, co-surfactant, and oil that are present in the nanoemulgel act as enhancers for permeation. Studies for stability were performed at the refrigerator and normal temperatures which indicated the Babchi oil formulation was stable and there were no observed changes in pH or the content of the components in the formulation. These factors during the study provided an improved illustration of drug stability and the effect of Babchi oil. Therefore, this study provides sufficient data to provide evidence for the use of Babchi oil nanoemulgel in the treatment use of psoriasis.

## 3. Conclusions

Babchi oil nanoemulgel has appropriate viscosity and was recently created for transdermal administration. The interaction between skin and nanoemulgels influences the rhamnolipid with incredibly precise penetration and enhances its potential, contributing significantly to drug absorption through the skin. In comparison to conventional pharmaceuticals, this Babchi oil-modified nanoemulgel demonstrated a significant improvement in drug penetration, showing that these nanoemulsion systems are possible carriers or vehicles for surrogate Babchi oil delivery.

## 4. Materials and Methods

### 4.1. Materials

Babchi oil and rhamnolipid along with Propylene glycol was obtained from Sigma Merck. Other materials such as ethanol and required chemicals were also purchased from Sigma Merck. The reagents used in the study were analytically graded. 

### 4.2. Experimental Methods

#### 4.2.1. Solubility of Babchi Oil

The solubility of Babchi oil in surfactants (rhamnolipid) and co-surfactants (propylene glycol) was observed by dissolving the oil within these in excessive amounts. To achieve equilibrium, the sample was constantly swirled for ten minutes in a vortex mixer and preserved at room temperature in an isothermal shaker for seventy-two hours. The samples that were equilibrated were then centrifuged at 3000 rpm for fifteen minutes. The supernatant acquired underwent filtration from a 0.45 μm filter membrane and was diluted using the mobile phase. The content of the drug was determined by using a UV-VIS spectrophotometer of Shimadzu-1700, Japan set at 260 nm. 

#### 4.2.2. Screening of Nanoemulsion Components

Based on solubility experiments, Babchi oil has the best solubilization ability. Surfactant and co-surfactant screening were performed using the percentage of transmittance. The capacity of surfactants (rhamnolipid) to emulsify was tested by the addition of 300 mg into the Babchi oil. To accomplish homogeneity, the sample was carefully heated at forty to forty-five degrees Celsius for thirty seconds. In order to formulate a fine emulsion, a measurement of 50 mg of the sample was taken and then blended with 50 mL of double distilled water. The overall turbidity of the final sample was assessed visually. After two hours, transmittance tests through the UV-VIS spectrophotometer of Shimadzu-1700, Japan were performed on the emulsions at 700 nm and a blank of water with double distillation as a narrow spectrum focused more on particles with shorter wavelengths. Out of a variety of co-surfactants that were screened, propylene glycol was used for the nanoemulsion formulation as it is considered safe. Propylene glycol (mg), rhamnolipid (mg), and Babchi oil (mg) formulations were created and analyzed in a similar method as indicated in the screening technique of surfactants. 

#### 4.2.3. Construction of Phase Diagrams

Titration methods were used to construct diagrams for pseudoternary phases. To create the surfactant, co-surfactant, and oil phase we used rhamnolipid, propylene glycol, and Babchi oil. Ratios of surfactant and co-surfactant weights that were used in the study were 2:1, 1:1, and 1:0 and underwent optimization to determine the exact ratio at which maximum area for nanoemulsion existence could be achieved. 

These formulations underwent titration in presence of water through micro syringing drops until the separation of phase or turbidity began. Within the other batch, similar ratios of surfactant, co-surfactant, and water were made and parameters were visualized. The formulations were briskly agitated for long enough periods for homogenization to occur in both situations. The endpoints of the formulations were monitored visually through a darkened screen by lighting the formulations using white light. To ensure repeatability, the tests were carried out thrice. The percent of mass components of Babchi oil and the combination of surfactant and co-surfactant along with the water was determined and represented on coordinates in the triangular form to build the diagrams for the pseudoternary phase from the endpoint. 

#### 4.2.4. Nanoemulsion Formulation

Babchi oil was added to the samples of surfactant and co-surfactant with different ratios that were observed from diagrams of the pseudoternary phase. Drop by drop, an adequate quantity of water was poured into the sample. The Babchi oil nanoemulsion was thus created by constantly stirring the formulation at the temperature of the room. The nanoemulsions that were prepared were kept at room temperature for future research.

#### 4.2.5. Nanoemulsion Optimization

Optimization of nanoemulsion was performed through 3 levels and 3-factor software of Design expert, namely Box–Behnken version 12 design of Stat-Ease, USA. The impacts of several processing factors such as concentration, sonication, zeta potential, size of particles, and efficiency of entrapment along with the in vitro release of nanoemulgel were comprehensively examined. The different levels used were observed with axial-α, low, medium, high, axial + α of oil and Smix with dependent variables to be particle size, zeta potential (ZP), and entrapment efficiency. Table 3 depicts the variables used during the CCRD to optimize the formulation. The design included a formulation run with several combinations at three points of center to evaluate the impact of the variables. Several models are provided by polynomial equations along with response plots for the surface to assess the influence of different variables and factors such as quadratic and linear factors. Of all the models, both individual and combined influence on dependent variables was best demonstrated by the quadratic model. 

##### Nanoemulsion Structure and Morphology

TEM (Transmission electron microscope) of Hitachi H7500, Japan was used to evaluate the shape and small structures of loaded nanoemulsion drugs. Water was used to dissolve the nanoemulsion compositions in a 1:10 ratio. Thereafter, a dissolved nanoemulsion droplet was implemented on the holey grid of film, dyed, and dried with a 1% aqueous solution of phosphotungstic acid.

##### Nanoemulsion Micromeritics 

Dynamic light dispersion with zeta-sizer HSA 3000 was used to analyze the size of globules of the nanoemulsion and index of polydispersity (PDI) of Malvern Instruments Ltd., UK. Before determining the PDI and size of globules, all the formulations were sonicated.

##### Nanoemulsion Conductivity and Viscosity 

At twenty-five degrees, the electrical conductance of the nanoemulsion was measured through an EC Testr 11+, USA conductance meter. This test was carried out thrice for reproducibility. 

The nanoemulsion prepared was observed for viscosity using the viscometer of Brookfield DV-II+ Pro without dilution. The formulation was placed within the beaker for five minutes before being measured at 0.5, 1, 2.5, and 5 rpm with a spindle. The accompanying dial was read on and recorded from the viscometer. 

##### Nanoemulsion-Based Hydrogel (Nanoemulgel) Formulation

Nanoemulgel was formed when each of the formulations was observed to be nanosized and so integrated inside the matrix of gel. The matrix of the gel base was chosen to be carbomer 940. The phase for oil was created by combining Babchi oil, rhamnolipid, and propylene glycol. Swelling of carbomer 940 was performed in a small amount of water for twenty-four hours to generate solutions of high viscosity. During this duration, the oily phase was progressively added into the viscous carbomer 940 formulations during the magnetic stir. After adjusting the pH from 6 to 9 using triethanolamine, nanoemulgel was produced. 

#### 4.2.6. Nanoemulsion Characterization

##### Particle Size and Polydispersity Index (PDI)

Rhamnolipid as surfactant and propylene glycol as cosurfactant was mixed with distilled water to produce an O/W type of nanoemulsion with Babchi oil. The nanoemulsion was prepared and the combination was sonicated for twenty minutes in an ice bath with the usage of a sonicator of 20 kHz with a peak power of 750 Watts. To detect the diameter of the mean droplet, the Litesizer-500 particle size analyzer was used with PDI.

##### Drug Content Determination

By dissolving 100 mg of the created nanoemulgel in 10 mL of distilled water, the quantity of medication included in the nanoemulgel was evaluated. This combination was tested using a UV spectrophotometer at 260 nm against distilled water as a blank control. It was then quantified by spreadability.

The drug content was determined by the following formula:

Concentration = Absorbance/(E1 cm1%) × Dilution factor × 10.

##### pH Determination

As the formulation would be administered to the skin, pH monitoring was required to guarantee that it was non-irritating. A digital pH meter was used to analyze the formulation pH and was measured at room temperature.

##### Spreadability

The potential spreading capacity of the nanoemulgels was measured 48 h after preparation. The spreadability was measured by evaluating the nanoemulgel diameter after spreading it across two glass plates for one minute. The mass of nanoemulgel was positioned upon the glass slide with one a pre-marked circle diameter of one centimeter, and a second glass slide had been placed. The diameter was observed to be grown as a result of the masses introduced, causing the gels to spread. The formulation may be used to calculate spreadability:

S = (m · l)/t.

The initial S is the ability to spread, m is the higher slide weight, l is the upper slide length, and t is the required time. 

##### Measurements of Viscosity and Rheological Management

The produced preparations were evaluated for viscosity through spindle number 4 of Brookfield DV-II+ Pro viscometer at various angular speeds at 31.0 ± 0.1 °C. The rheological behavior of the formulation of nanoemulgel was assessed using plate and cone configurations of a 40 mm cone with a 2.5-degree cone angle. Rheology experiments were carried out at 25 °C with shear rates ranging from 53.21–496.5 s^−1^.

The power law equation was used to determine the consistency and flow indexes:

Ʈ = *Krn*.

The symbol Ʈ refers to shear stress, *r* refers to shear rate, *K* refers to the index of consistency, and *n* is the index of flow.

Log analysis on both sides is expressed as follows:

Log Ʈ= log K+ n log r.

Therefore, the slope of the graph of logs of shear stress versus logs of shear rate was selected as the flow index, while the inverse function of the Y-intercept provided the index for consistency.

#### 4.2.7. Ex Vivo Drug Studies on Permeation

Ex vivo studies for permeation were conducted using the Franz cell for diffusion, which is a proven approach for predicting the delivery of drugs through the skin. The skin of Wistar rats was removed for this research. The sacrificed rats’ hair from their dorsal part was detached using a surgical blade of 24 numbering in the region from tail to head. The shaved skin was split, and unwanted fat along with the connective tissues was excluded with a scalpel. The skin was further removed and cleaned with saline and inspected for its integrity to be used. The skin of the rats was placed on an assembly cell with a 10 cm^2^ high area for diffusion, with the stratum corneum facing the donor segment and the dermal side facing the receiver segment.

#### 4.2.8. Determination of Nanoemulgel Drug Content

Through the lower back direction using 24 surgical blades, the animal was sacrificed through the dorsal side. A knife was used to remove superfluous fat and connective tissue from the shaved area of the animal skin. The removed skin was cleaned with normal saline before being inspected for integrity and then utilized. 

#### 4.2.9. DPPH Scavenging Activity of Nanoemulgel

The DPPH (2,2 diphenyl-1-picryl hydrazyl) technique developed by Williams et al. was used to assess the overall radical scavenging activity of Babchi oil emulsions before and following encapsulation [31]. The donating capacity of electrons of the anti-oxidants causes the violet hue of the solution to become colorless at normal temperature. Upon dissolution of the sample of 0.5 within methanol of 3 mL, the resultant solution was processed using the methanolic solution of 0.3 mL for DPPH. During the progression of the reaction, this formulation was held inside a darkened room for an hour. The changes in the color indicated the properties of antioxidants in the formulation due to the donating capacity of hydrogen. The sample of 0.3 mL and 3.3 mL of methanol was included within the blank while 0.3 mL of DPPH reagent along with 3.5 mL of methanol was included in the control. At 517 nm, the formulations were examined in a spectrophotometer. 

#### 4.2.10. In Vitro Release and Permeation Studies

The process of dialysis bag was used to examine the in vitro drug release from conventional and Babchi oil nanoemulgels. The dialysis bags that were activated beforehand were carefully secured after adding 1 mL of the conventional and similarly 1 mL Babchi oil into the gel. The bag of dialysis was then immersed in a 200 mL medium of phosphate buffer along with methanol for dissolving at pH 6.8. This was maintained at room temperature and stirred at 400 rpm. At preset intervals, HPLC (High-Performance Liquid Chromatography) was used to evaluate all the formulations. Different models such as the zero-order release model, first-order release model, Higuchi model, and Korsmeyer Peppas model were used for analysis. The total quantities of Babchi oil released from the membrane were plotted as diffusion areas per time. 

#### 4.2.11. Release of Babchi Oil Nanoemulgel

The process involved entailed replacing a dialysis bag with the rat skin section. Any extra hairs were removed from the rat skin and were washed with a solution of Tyrode. These skin segments were immersed in 1 mL of Babchi oil nanoemulgel and conventional formulation. The study was conducted with the placement of applied rat skin in 100 mL of solution of Tyrode at room temperature with constant agitation on Hanson Research SR8 plus of California, USA at the speed of 100 rpm. This was observed at various time points, and a sample of 2 mL was removed and a quantity similar to the solution of Tyrode was included as a preservative. The Babchi oil concentration was observed at 218 nm through a spectrophotometer and the procedures were repeated thrice for reproducibility.

#### 4.2.12. Dermatokinetic Studies

Application of Babchi oil nanoemulgel on rat skin was observed with Franz Diffusion Cell (FDC) according to the literature search on in vitro skin permeation studies. Using the tool, we analyzed the content and concentration of Babchi oil and its formulated mixture at different periods of 0, 0.5, 1, 1.5, 2, 3, 4, 5, 6, 7, and 8 hours with the entire skin obtained from FDC. Excess nanoformulation was removed from the skin and rinsed using saline of pH 7.4. It was then dipped in the mildly warm water of temperature 60 °C for three minutes. We then separated the dermis and epidermis layers of skin using forceps. These layers were cut into small parts and kept in methanol (5 mL) for a day to obtain the Babchi oil content. The solution of methanol left after removing the layers underwent membrane filtration and the Babchi oil content was measured through HPLC. Separate concentrations per cm^2^ of Babchi oil from dermis and epidermis layers were observed with time and parameters of T skin max, C skin max, AUC 0–8 h, and Ke were analyzed.

#### 4.2.13. Stability Studies

Assessment of stability of the created formulation was performed by keeping it for three months at different temperatures of 30 ± 2 °C and 40 ± 2 °C with a humidity level of 60 ± 5%. According to the Iqubal et al. method, the samples were observed at different intervals of 0, 1, 2, and within 3 months to observe their appearance, separation of phases, size of globules, EE and PDI. These measurements were done thrice to determine the repeatability [32].

#### 4.2.14. Statistical Analysis

The measurements obtained for the experiment were in triplicate values and were presented in values of mean ± SD (standard deviation). The analysis of statistical difference was performed on the flux at the stable stage and the permeation of ex vivo on intervals that were pre-determined from the formulations. Unpaired *t*-test was utilized with a *p*-value < 0.05 as the significance level.

## Figures and Tables

**Table 1:** Responses obtained using CCRD.

Run	Oil % *v*/*v*	S_mix_ % *v*/*v*	Particle Size (nm)	Zeta Potential	EE (%)
1	3	5	193.43	−22.57	89.43
2	4	6	249.35	−16.36	89.11
3	3	5	194.34	−23.13	88.09
4	4	4	283.13	−25.08	90.07
5	1.5	5	120.34	−13.98	58.21
6	3	5	193.25	−22.98	88.61
7	3	5	194.12	−23.62	89.75
8	2	6	131.87	−14.87	65.98
9	3	6.4	176.21	−12.76	79.64
10	4.4	5	302.45	−18.25	93.12
11	3	3.5	204.54	−29.08	76.12
12	3	5	195.04	−22.11	88.04
13	2	4	144.04	−24.13	66.31

**Table 2:** Dermatokinetic study chart.

Skin Part	Type	T_max_	C_max_	AUC	Ke
Epidermis	Formulation	2	220.049	872.4898	0.13295
	Conventional	2	114.569	472.9198	0.137736
Dermis	Formulation	2	164.875	644.4886	0.139326
	Conventional	2	375.918	375.9179	0.139155

**Table 3:** Variables used in CCRD to optimize the formulation.

Factors	Levels Used				
Independent Variable	Axial −α	Low (−1)	Medium (0)	High (+1)	Axial + α
A—Oil (% *v*/*v*)	1.58	2	3	4	4.41
B—S_mix_ (% *v*/*v*)	3.58	4	5	6	6.41
Dependent Variable	Constraints used				
R1—Particle size (nm)	Minimum				
R2—ZP	Maximum				
R3—Entrapment efficiency (%)	Maximum				

## Data Availability

Not applicable.
